# Diagnostic performance of serum pentraxin-3 in pediatric acute appendicitis: a prospective diagnostic validation study

**DOI:** 10.1007/s00383-022-05289-7

**Published:** 2022-12-01

**Authors:** Javier Arredondo Montero, Giuseppa Antona, Mónica Bronte Anaut, Carlos Bardají Pascual, Raquel Ros Briones, Amaya Fernández-Celis, Adriana Rivero Marcotegui, Natalia López-Andrés, Nerea Martín-Calvo

**Affiliations:** 1 Pediatric Surgery Department, Hospital Universitario de Navarra, Calle Irunlarrea 3, 31008 Pamplona, Navarra Spain; 2 School of Medicine, University of Navarra, Pamplona, Navarra Spain; 3 Pathology Department, Hospital Universitario de Araba, Vitoria, Basque Spain; 4 Cardiovascular Translational Research, NavarraBiomed (Miguel Servet Foundation), Hospital Universitario de Navarra, Universidad Pública de Navarra (UPNA), IdiSNA, Pamplona, Spain; 5 Clinical Analysis Department, Hospital Universitario de Navarra, Pamplona, Spain; 6 Department of Preventive Medicine and Public Health, School of Medicine, University of Navarra, Pamplona, Navarra Spain; 7 Instituto de Investigación Sanitaria de Navarra (IdiSNA), Pamplona, Navarra Spain; 8 CIBER Fisiopatología de la Obesidad y Nutrición (CIBERobn), Health Institute Carlos III, Madrid, Spain

**Keywords:** PTX3, TSG14, Pediatric acute appendicitis, Complicated, ROC, Diagnosis

## Abstract

**Introduction:**

Pediatric acute appendicitis (PAA) is a pathology with a high rate of diagnostic error. The search for new diagnostic tools is justified by the high morbidity and healthcare costs associated with diagnostic error.

**Methods:**

We designed a prospective study to validate serum pentraxin-3 (PTX3) as a diagnostic tool in PAA. Participants were divided into three groups: (1) patients with no underlying pathology (2) patients with non-surgical abdominal pain and (3) patients with a confirmed diagnosis of PAA. For further analyses, patients in group 3 were divided into complicated or uncomplicated PAA. Quantitative variables were expressed as medians and interquartile ranges and categorical variables as percentages. Quantitative variables were compared using the Kruskal–Wallis test and the Mann–Whitney *U* test. Diagnostic performance was evaluated with ROC curves.

**Results:**

This study included 215 patients divided into group 1 (*n* = 63), group 2 (*n* = 53) and group 3 (*n* = 99). Median serum PTX3 values were 2.54 (1.70–2.95) ng/mL, 3.29 (2.19–7.64) ng/mL and 8.94 (6.16–14.05) in groups 1, 2 and 3, respectively (*p* = 0.001). Patients with complicated PAA showed significantly higher values than patients with uncomplicated PAA (*p* = 0.04). The AUC (group 2 vs. 3) was 0.77 (95% CI 0.69–0.85) and the best cut-off point was at 7.28 ng/mL, with a sensitivity of 61.3% and a specificity of 73.1%. The AUC (complicated vs. uncomplicated PAA) was 0.65 (95% CI 0.54–0.77) and the best cut-off point was 12.33 ng/mL, with a sensitivity of 51.72% and a specificity of 72.73%.

**Conclusions:**

The diagnostic ability of serum PTX3 in PAA is only moderate and therefore it cannot be considered a definitive diagnostic test. The discriminatory ability of PTX3 between complicated and uncomplicated PAA is poor. These findings, which contrast with those reported to date, should be validated with future properly designed prospective studies.

**Supplementary Information:**

The online version contains supplementary material available at 10.1007/s00383-022-05289-7.

## Introduction

Pediatric acute appendicitis (PAA) is the most frequent urgent pediatric abdominal pathology requiring surgical intervention. Currently, diagnosis is based on physical examination and clinical history, and the complementary diagnostic studies of choice are the basic laboratory tests (blood count and biochemistry) and the abdominal ultrasound [1]. Given the important social, economic and health repercussions of misdiagnosis, multiple diagnostic tools have been explored in recent decades in the context of PAA. On one hand, ratios based on the usual parameters of the hemogram have been proposed, such as the neutrophil–lymphocyte ratio and the platelet–lymphocyte ratio [2, 3]. Scores based on clinical, analytical and radiological variables have also been explored [4, 5]. On the other hand, new biomarkers have been evaluated prospectively in serum [6], urine [7], faeces [8] and saliva [9].

Pentraxins constitute a family of multimeric pattern-recognition proteins highly conserved in evolution and with a recognized role in innate immunity. A well-known and illustrative example is Pentraxin-1, a short pentraxin, commonly known as C-reactive protein or CRP. Pentraxin-3 (PTX3), also known as tumor necrosis factor (TNF)-inducible gene 14 protein or TSG-14, is a long pentraxin encoded by the PTX3 gene, located on chromosome 3 [10]. It is known to be produced and secreted by multiple cell populations (dendritic cells, fibroblasts and endothelial cells among others) in response to primary inflammatory signaling, such as those associated with TNF alpha, interleukin (IL)-1 beta and toll-like receptor (TLR). At the biological level, this molecule behaves as an acute phase reactant, significantly increasing its values in inflammatory and infectious processes [11]. Its role has been studied in multiple pathologies, including several neoplasms [12], coronary syndromes [13] and SARS-CoV-2 infection [14]. The evidence regarding its diagnostic utility in PAA is still building and recent studies showed promising results [15–18], especially to discriminate between complicated and non-complicated PAA [19].

## Materials and methods

This study was approved by our center's clinical research ethics committee on December 18, 2020, under code PI_2020/112. The ethical principles of the Declaration of Helsinki were followed during the conduct of this study. Parents or legal guardians of all participants signed an informed consent prior to the inclusion in the study.

### Study design

This study, which belongs to the BIDIAP cohort [20, 21], is a prospective, observational study to determine the diagnostic performance of serum PTX3 in PAA. Three groups of pediatric patients were included in this study: (1) patients with no underlying pathology who underwent scheduled outpatient surgery, (2) patients with acute abdominal pain who presented to the emergency department with a suspected diagnosis of acute appendicitis and in whom the diagnosis was finally excluded—also defined as non-surgical abdominal pain (NSAP), and (3) patients with histologically confirmed diagnosis of acute appendicitis. For further analysis, patients in group 3 were stratified in uncomplicated PAA (congestive, phlegmonous or suppurative appendicitis) or complicated PAA (gangrenous or perforated appendicitis). Sociodemographic, clinical, analytical, surgical, radiological and histological data of all patients were extracted from participants’ clinical records.

Patients were recruited when the personnel conducting the investigation were available at the center. The recruitment period extended from February to December 2021. Inclusion and exclusion criteria are shown in Supplementary file 1.

All patients in group 2 were contacted 2 weeks after their inclusion in the study to confirm that they had not been diagnosed with PAA in that period. All patients in group 3 were reviewed on an outpatient basis in the first postoperative month.

### Sample collection and measurement of pentraxin-3 levels

A venous blood sample was taken from each patient in a vacutainer tube with separator gel (3.5 mL). In patients in group 1, this sample was taken prior to the intervention. In patients in groups 2 and 3, it was taken at the time of inclusion in the study, during their stay in the emergency department. All serum samples were taken before starting empirical antibiotic treatment.

Serum samples were frozen and processed by laboratory personnel blinded to the patient’s group. Determinations were made by a commercial ELISA following the manufacturer's instructions (R&D systems). C-reactive protein (CRP) and procalcitonin (PCT) were determined by immunoassay on an Alinity-CI analyzer (Abbott). All the markers were measured in the same sample concurrently.

### Statistical analysis

For descriptive purposes, median and interquartile ranges were used for quantitative variables and proportions for categorical variables. Kolmogorov–Smirnov test was used to assess the normality of quantitative variables. Sociodemographic and clinical variables were compared between groups using the Kruskal–Wallis test and the Mann–Whitney U test. To calculate the discriminative capacity of the PTX3, we calculated the area under the receiver operating characteristic curves (ROC). Additionally, the distance on the ROC curve of each PTX3 value was calculated as the square root of [(1 − sensitivity)^2^ + (1 − specificity)^2^]. The PTX3 value with the shortest distance on the ROC curve was considered the optimal cut-off.

To compare the discriminatory ability of PTX3 with that of other routine serum markers, we calculated the AUC, sensitivity and specificity for leucocytes, neutrophils, CRP and PCT. Statistical significance was settled in a *p* value < 0.05. Statistical analysis was performed with STATA 17.0 (StataCorp LCC).

## Results

### Demographic and clinical characteristics

This study included 215 patients, divided into 3 groups: (1) patients who underwent scheduled major outpatient surgery (*n* = 63), (2) patients with non-surgical abdominal pain in whom the diagnosis of PAA was excluded (*n* = 53) and (3) patients with a confirmed diagnosis of PAA (*n* = 99). Group 3 was further subdivided into complicated PAA (*n* = 31) and uncomplicated PAA (n = 68). Twelve participants were excluded from the analyses due to the lack of serum sample at the time of diagnosis (group 1 = 6 patients; group 2 = 1 patient; group 3 = 5 patients). No statistically significant differences were found between the 12 excluded patients and the 203 included patients. Participants’ sociodemographic characteristics by group are shown in Table 1. Significant differences between groups were observed for age, sex, weight and body mass index.

**Table 1:** Sociodemographic characteristics of the study patients

Sociodemographics	Group 1 (ambulatory controls) *N* = 57	Group 2 (NSAP) *N* = 52	Group 3 (PAA) *N* = 94	Total	*p* value
Age (years)	8.63 (3.26)	11.09 (2.48)	9.55 (3.03)	9.69 (3.09)	< 0.001
Sex (male/female) (%)	46/11 (80.70%)	24/28 (46.15%)	60/34 (65.30%)	130/73 (64.03%)	0.001
Height (centimeters)	1.45 (0.23)	1.50 (0.15)	1.41 (0.18)	1.44 (0.18)	0.22
Weight (kilograms)	35.58 (18.25)	45.36 (15.40)	35.61 (12.02)	38.11 (15.41)	< 0.001
Body mass index (kg/m^2^)	21.52 (6.83)	20.45 (3.97)	17.26 (2.27)	18.75 (3.80)	< 0.001

Numbers are mean (standard deviation) or number (percentage)

Compared with patients in group 2, those in group 3 had significantly more emetic episodes (Table 2).

**Table 2:** Clinical characteristics and serum biomarker values of groups 2 (NSAP) and 3 (PAA)

Clinical variables	Group 2 (NSAP) N=52	Group 3 (PAA) N=94	*P* value
Hours of pain evolution	31.58 (23.13)	27.43 (19.85)	0.40
Fever > 37.8 (yes/no/missing) (%)	15/37 (28.84%)	30/63/1 (31.91%)	0.41
Number of diarrheal stools	0.40 (1.21)	0.66 (2.46)	0.63
Urinary symptoms (yes/no/missing) (%)	8/44 (15.38%)	21/72/1 (22.34%)	0.21
Number of emetic episodes	0.56 (1.96)	2.51 (2.46)	< 0.001
Hyporexia (yes/no/missing) (%)	35/15/2 (67.30%)	73/17/4 (77.65%)	0.15
Leucocytes (1 × 10^9/L)	10.17 (3.37)	16.17(4.82)	< 0.001
Neutrophils (1 × 10^9/L)	6.68 (3.59)	13.14 (4.75)	< 0.001
CRP (mg/L)	17.35 (31.42)	46.37 (54.92)	< 0.001
PCT (ng/mL)	0.11 (0.23)	1.34 (5.60)	< 0.001
PTX3 (ng/mL)*	3.29 (2.19–7.64)	8.94 (6.16–14.05)	< 0.001

Numbers are mean (standard deviation) or numbers (percentage)

*Median, Interquartile range

### Serum biomarkers

Significant differences were also observed between groups 2 and 3 in analytical variables, with higher levels of leucocytes, neutrophils, CRP, PCT and PTX3 in patients in group 3 (Table 2).

**Fig. 1:**
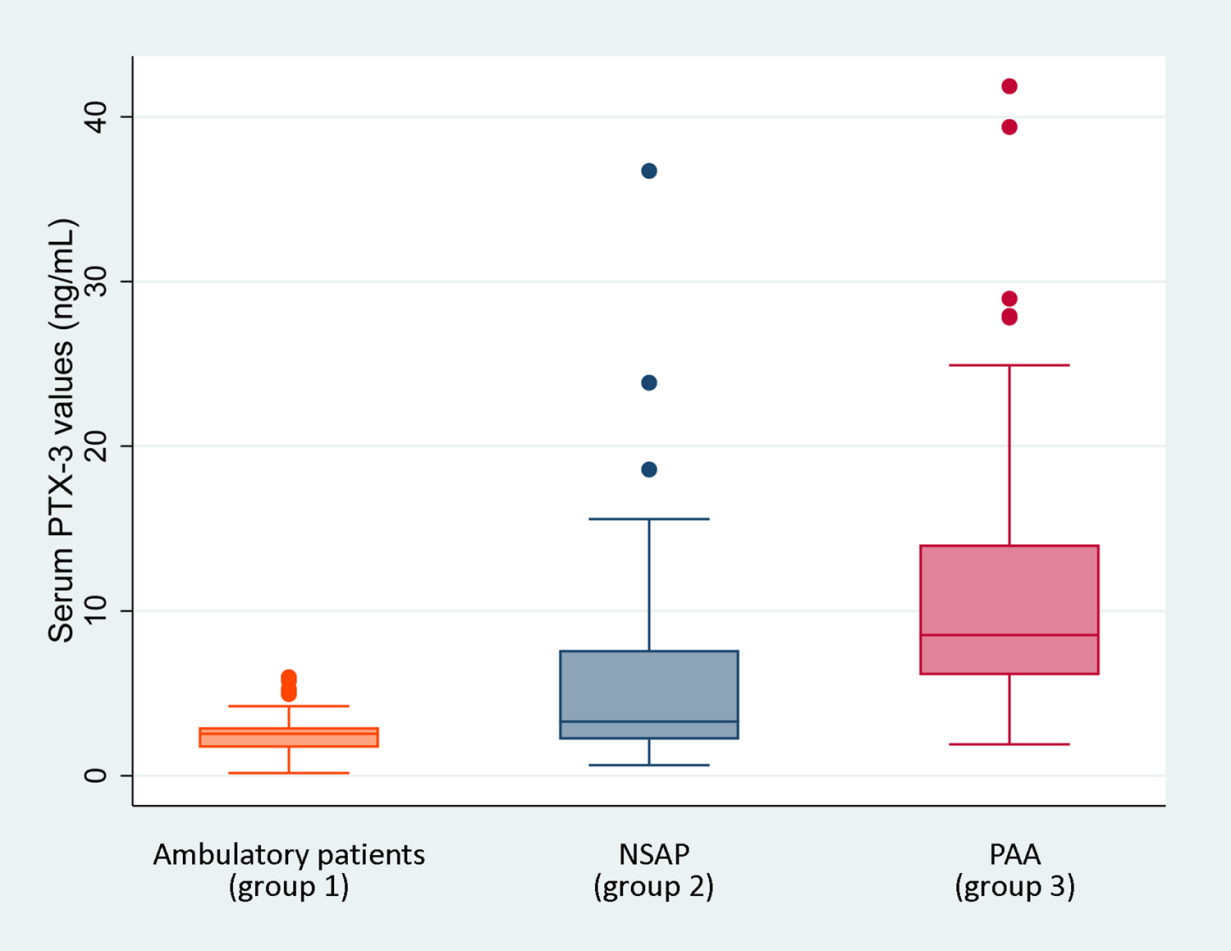
Box-plot representation of PTX3 values in the different study groups

Median (IQR) serum PTX3 values were 2.54 ng/mL (1.70–2.95) in group 1, 3.29 ng/mL (2.19–7.64) in group 2, and 8.94 ng/mL (6.16–14.05) in group 3 (*p* = 0.001). The graphical representation of the PTX3 serum levels by groups is shown in Fig. 1.

Regarding the capacity of PTX3 to discriminate between patients from groups 2 and 3 we found an AUC of 0.77 (95% CI 0.69–0.85) (*p* > 0.001). The cut-off point with the shortest distance on the ROC curve resulted in 7.28 ng/mL, with a sensitivity of 61.3% and a specificity of 73.1%. Higher value of AUC was observed for the discrimination between patients in groups 1 and 3 (AUC: 0.95; 95% CI 0.92–0.982) (*p* < 0.001). In this analysis, the cut-off point was established at 5.94 ng/mL, resulting in a sensitivity of 78.5% and a specificity of 98.9%. In further analysis, we calculated the AUC for the discrimination between patients from groups 1 and 2 vs. patients in group 3, obtaining an AUC of 0.86 (95% CI 0.81–0.91) (*p* < 0.001). In this analysis, the cut-off point was established at 5.34 ng/mL, resulting in a sensitivity of 81.7% and a specificity of 78.9%.

Table 3 shows the AUC values, sensitivity and specificity of the different serum markers analyzed in this study for the comparison of patients in group 2 versus patients in group 3. The biomarkers with the highest discriminatory capacity were leucocytes (AUC 0.84; 95% CI 0.78–0.91) and neutrophils (0.84; 95% CI 0.77–0.90), followed by PTX3 (AUC 0.77; 95% CI 0.69–0.85). Nevertheless, compared with the other biomarkers, PTX3 showed the lowest value of sensitivity at the given cut-off point.

**Table 3:** Diagnostic performance of the different serum markers analyzed in the study

Serum biomarker	AUC (Group 2 vs 3)	95% CI	Proposed cut-off	Sensitivity (%)	Specificity (%)
Leucocytes (1 × 10^9/L)	0.84	(0.78–0.91)	11.1	85.57	70.59
Neutrophils (1 × 10^9/L)	0.84	(0.77–0.90)	11.4	84.54	74.51
CRP (mg/L)	0.73	(0.65–0.82)	1.1	91.75	47.17
PCT (ng/mL)	0.69	(0.59–0.79)	0.03	84.52	58.70
PTX3 (pg/mL)	0.77	(0.69–0.85)	7.28	61.3	73.1

*AUC* area under the curve

Additionally, we analyzed alternative cut-off values to evaluate their diagnostic performance in terms of sensitivity, specificity and positive likelihood ratio (Table 4). As expected, sensitivity decreased and specificity increased as the cut-off point raised. The highest percentage of correctly classified participants was observed for the cut-off of 2.77 ng/mL, which showed high sensitivity (96.77%) but a rather low value of positive likelihood ratio (1.62).

**Table 4:** Proposed alternative cut-offs for serum PTX3 (group 2 vs group 3)

PTX3 cut-off value (pg/mL)	Positive likelihood ratio	Correctly classified (%)	Sensitivity (%)	Specificity (%)
2.00	1.14	68.3	98.9	13.46
2.77	1.62	76.55	96.77	40.38
4.13	1.91	75.9	88.2	53.85
7.28	2.31	66.2	61.3	73.1
11.84	5.03	57.9	38.7	92.3

In further analysis, we compared serum values of different biomarkers between the uncomplicated PAA and complicated PAA group (Table 5). Significant higher values were observed in the patients with complicated PAA for leucocytes, neutrophils, CRP, PCT and PTX3. Finally, in the analysis aimed to evaluate the capacity of PTX3 to discriminate between complicated and uncomplicated PAA, we obtained an AUC of 0.65 (95% CI 0.54–0.77) and a cut-off point of 12.33, which associated a 51.72% sensitivity and a 72.73% specificity.

**Table 5:** Serum biomarker values (complicated PAA vs non-complicated PAA)

Serum biomarker	Non-complicated PAA (*n* = 65)	Complicated PAA (*n* = 29)	*p* value
Leucocytes (1 × 10^9)	15.25 (12.15−17.75)	18.4 (15.7−21.8)	0.002
Neutrophils (1 × 10^9)	12.2 (9−15.25)	15.3 (13−17.6)	0.001
CRP (mg/L)	19.6 (5.45−40.3)	63.3 (17.4−108)	0.001
PCT (ng/L)	0.05 (0.03−0.15)	0.48 (0.20−1.91)	< 0.001
PTX3 (pg/mL)	7.59 (5.22–12.89)	12.32 (7.41–15.60)	0.04

Values expressed as medians (interquartile range)

**Fig. 2:**
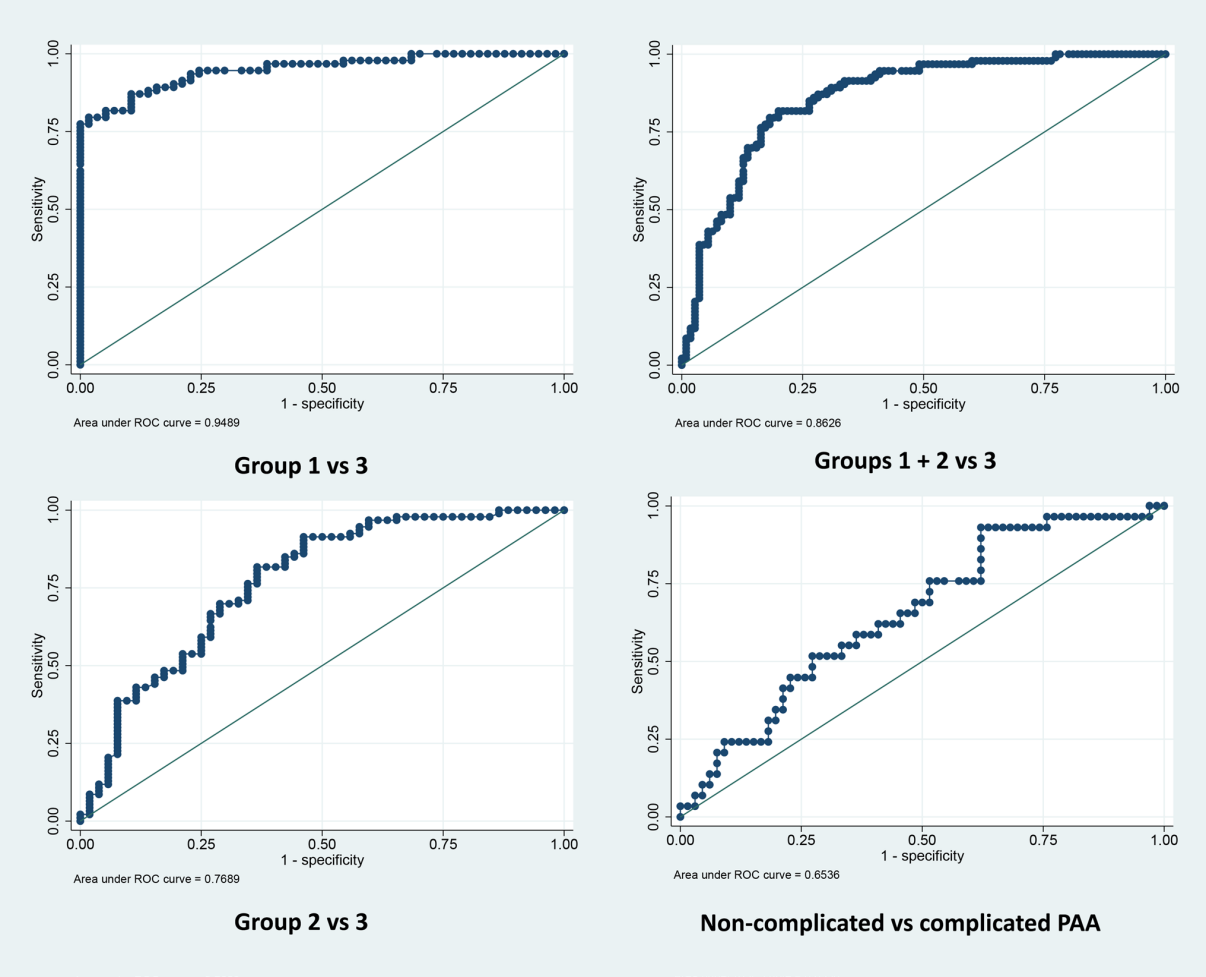
Graphical representation of the PTX3 ROC curves. Above, left: logistic regression (groups 1 vs 3). Above, right: logistic regression (groups 1 + 2 vs 3). Bottom, left: logistic regression (groups 2 vs 3). Bottom, right: logistic regression (non-complicated vs complicated PAA groups)

The graphical representation of the different ROC curves is shown in Fig. 2.

## Discussion

In this prospective study, we evaluated the diagnostic performance of serum PTX3 for the diagnosis of PAA. After the different comparative analyses, we obtained an AUC value of 0.77 for the comparison of the NSAP and PAA groups, and an AUC value of 0.86 for the comparison of the NSAP + ambulatory control groups and the PAA group. These results translate into a moderate diagnostic yield for serum PTX3 in the context of PAA. On the other hand, the capacity of PTX3 to discriminate between complicated and uncomplicated PAA was poor. An AUC of 0.65 is insufficient to consider it a promising diagnostic test in clinical practice since it does not exceed the discriminatory capacity of other routine tests, such as physical examination, blood count, abdominal ultrasound or even recently evaluated biomarkers such as serum IL-6. [21]

Our findings contrast with those published in previous research. To date, the diagnostic performance of serum PTX3 in PAA has been evaluated in three studies [15–17]. The first study [17], published in 2019, had a sample size of 88 patients, divided into three groups: 28 healthy controls, 26 patients with NSAP and 34 patients who underwent appendectomy. Mean ± SD serum values of PTX3 were 1.09±0.97 ng/mL, 4.07±1.55 ng/mL and 12.82±4.96 ng/mL for healthy controls, NSAP patients and patients who underwent appendectomy respectively. The proposed cut-off was 5.6 ng/mL and the AUC was 0.98 (sensitivity 91.8%, specificity 90.7%). The second study, published in 2020 [16], had a sample size of 55 patients (15 healthy controls and 40 patients in the PAA group, subdivided into perforated and non-perforated PAA). The median (range) serum values of the healthy controls group were 1.01 (0.82–1.28) ng/mL, the median (range) serum values of the perforated PAA group were 1.46 (1.05–23.42) ng/mL and the median (range) serum values of the non-perforated PAA group were 20.68 (1.02–28.47) ng/mL. The proposed cut-off was 1.30 ng/mL, and the AUC value was 0.94 (sensitivity 75% specificity 100%). The last study [15], published in 2020, had a sample size of 70 patients, divided into healthy controls (*N* = 8), patients with NSAP (*N* = 25) and patients with PAA (*N* = 37). The mean±SD serum PTX3 values were, respectively, 4.33±0.34 ng/mL, 6.88±2.93 ng/mL, and 14.35±7.32 ng/mL for healthy controls, NSAP patients and PAA patients respectively.. The cut-off value was set at 9.31 ng/mL and the AUC value at 0.83, with a sensitivity of 72.97% and a specificity of 88%. All working groups used different ELISA determination kits.

First, we believe that the results reported by two of the working groups [16, 17] may have overestimated the diagnostic performance of serum PTX3 in the diagnosis of PAA. AUC values above 0.95 indicate near-perfect diagnostic performance. In the case of the work of Ates et al.[16] this is related to the use of a control group composed exclusively of healthy volunteers. As in our work, the AUC is almost perfect in this scenario. It is noteworthy that in the work of Ates et al. [16] PTX3 values were found to be much higher in the non-perforated PAA group than in the perforated PAA group, which is less plausible from a biological point of view. Also, the same authors report very high AUC values for other common markers such as leukocytes (AUC = 0.96) [16], which are not consistent with the existing literature. Again, we attribute these findings to the use of a control group composed exclusively of healthy volunteers. We believe that, since the PTX3 is an acute phase reactant involved in innate immunity (and therefore, a molecule with a certain non-specific character), those values may be overestimated. We think the results reported by the Duman L et al. [15] may be closer to reality, and the differences with our study [AUC 0.83 vs 0.77 (95% CI 0.69–0.85)] may be, at least partially, explained by different statistical approaches.

While in our main analysis we opted for comparing specifically the NSAP group vs. the PAA group, in two of the three previous works [15, 17], the authors opted for merging the control groups (NSAP + ambulatory patients) when performing the ROC analysis. Given that the group of ambulatory control patients showed very low serum PTX3 values in all the studies (including ours), it is not surprising that previous studies reported higher differences between groups and, therefore, an overestimated discriminatory capacity of the PTX3. Indeed, we obtained a maximum AUC when we compared the PAA group vs the ambulatory patients group (AUC: 0.95; 95% CI 0.92–0.982) and it slightly decreased when we added to the control group the patient with non-surgical abdominal pain (AUC: 0.86; 95% CI 0.81–0.91). However, we believe that our main analysis is more realistic, since the diagnostic yield in routine clinical practice will be limited to patients with a suspected diagnosis of PAA (i.e., it will serve to discern between groups 2 and 3). Group 1 in our study served to delimit the baseline values of the molecule in a healthy population, but we do not believe it should be included the analysis, because it is not representative of the patients in whom PTX3 will be used to diagnose acute appendicitis in the clinical setting.

PTX3 is part of the pentraxin superfamily, of which C-reactive protein is one of the most important and well-known members. To date, this molecule has not been shown to be elevated specifically in gastrointestinal pathology, but rather appears to be a molecule that is elevated in the context of acute systemic inflammatory processes, including PAA. In our cohort, four participants in the group with non-surgical abdominal pain (7.5%) who presented serum values of PTX3 above the stablished cut-off (7.28 ng/mL) had microbiologically confirmed enteroinvasive diarrhea in the days following inclusion in the study. We believe this misclassification could have contributed to the fact that the discriminatory capacity of PTX3 in our study was lower than that observed in previous studies. The literature evaluating the diagnostic performance of PTX3 in other pediatric processes is scarce. To our knowledge, there are no studies that have evaluated PTX3 in gastrointestinal pediatric patholog beyond those mentioned here. Further studies are needed to better elucidate the changes in PTX3 in different inflammatory contexts in pediatric population.

In contrast with Ates et al. [16], we found significant higher mean serum levels of PTX3 in the group of patients with complicated PAA than in uncomplicated PAA. Considering the biological mechanism behind this association and taken into account that similar differences were observed for the rest of biomarkers that we analyzed, we believe that our findings may be closer to reality. However, we did not find any significant correlation between PTX3 serum values and time of evolution, unlike Duman L et al. [15], who reported a significant direct correlation between them.

It is worth mentioning that all previous studies have been carried out in Turkey. It is reasonable to think that there may be environmental and individual factors explaining the differences observed between our study and previous works. Indeed, even those studies presented a very wide range for PTX3 in both maximum and minimum serum values (from 0.82 to 28.47) and proposed cut-offs (from 1.30 to 9.31). Similar variability has been found in other serum biomarkers whose diagnostic performance in PAA has been analyzed [6, 8].

The aforementioned studies were recently evaluated in a systematic review and meta-analysis [22], but we believe that given the limitations previously described in relation to the studies included, and the limited sample size of these studies, the results of this review are limited and difficult to extrapolate.

Regarding efficiency, PTX3 is a time and cost-effective diagnostic tool. Each determination had an approximate cost of 10 euros and, although the processing time in our case was not evaluable, because the samples were frozen and processed in a deferred manner, the laboratory staff estimated that measurements could be obtained within 4 to 6 h after the sample extraction.

We acknowledge some limitations in our study. First, we used a convenience sampling which is susceptible of a selection bias. For a selection bias to be the explanation for our results, selection into the analytical sample would have to be related to the group (control or PAA) and PTX3 levels. More specifically, patients in the control group with high levels of PTX3 and PAA patients with low levels of PTX3 would have been less likely included in the analytical sample, which is unlikely and difficult to fully address given the available data. Due to the observational design of our study, we cannot deny the possibility of confounding by variables we did not account for, such as age and sex. Serum levels of some biomarkers are associated with age, but there is no previous evidence on an association between age and PTX3 levels. Matched design and stratified analysis are useful strategies for controlling for confounding, but our study had an independent design, and our sample size was insufficient subgroup analyses. Future studies should consider different strategies to assess the potential confounding effect of sociodemographic variables.

On the other hand, our study has several strengths, including a rigorous methodology and critical analysis of the data obtained in comparison with the existing literature and a large sample size, considerably larger than other series published to date. Another important strength is that the laboratory staff was blinded to patient’s group.

In conclusion, to date, no biomarker has shown sufficient discriminative capacity to be used as a single test for the diagnosis of PAA. According to our results, we do not believe that PTX3 could be used as a single marker for the diagnosis of PAA either. Further research is needed to elucidate whether the inclusion of PTX3 serum levels in a risk score that considers sociodemographic, clinical, and radiological variables could make a difference. Despite our findings, we believe that the study of potential new biomarkers for the diagnosis of PAA remains an open avenue for future studies.

### Supplementary Information

Below is the link to the electronic supplementary material.

Supplementary file1 (DOCX 16 KB)

## Data Availability

All data pertaining to this study are available upon justified request through the author in correspondence.
