# Correction: Diagnostic performance of serum pentraxin-3 in pediatric acute appendicitis: a prospective diagnostic validation study

**DOI:** 10.1007/s00383-022-05342-5

**Published:** 2022-12-13

**Authors:** Javier Arredondo Montero, Giuseppa Antona, Mónica Bronte Anaut, Carlos Bardají Pascual, Raquel Ros Briones, Amaya Fernández-Celis, Adriana Rivero Marcotegui, Natalia López-Andrés, Nerea Martín-Calvo

**Affiliations:** 1 Pediatric Surgery Department, Hospital Universitario de Navarra, Calle Irunlarrea 3, 31008 Pamplona, Navarra Spain; 2 School of Medicine, University of Navarra, Pamplona, Navarra Spain; 3 Pathology Department, Hospital Universitario de Araba, Vitoria, Basque Country Spain; 4 Cardiovascular Translational Research, NavarraBiomed (Miguel Servet Foundation), Hospital Universitario de Navarra, Universidad Pública de Navarra (UPNA), IdiSNA, Pamplona, Spain; 5 Clinical Analysis Department, Hospital Universitario de Navarra, Pamplona, Spain; 6 Department of Preventive Medicine and Public Health, School of Medicine, University of Navarra, Pamplona, Navarra Spain; 7 Instituto de Investigación Sanitaria de Navarra (IdiSNA), Pamplona, Navarra Spain; 8 CIBER Fisiopatología de la Obesidad y Nutrición (CIBERobn), Health Institute Carlos III, Madrid, Spain

**Correction: Pediatric Surgery International (2023) 39:27** 10.1007/s00383-022-05289-7

In the original publication, the unit of measurement for serum PTX3 in tables 3, 4, 5 was incorrect. In the original version it was pg/mL, and the correct unit is ng/mL.

